# Multistakeholder scientific advice for medicinal products used in combination with a medical device or a companion diagnostic in the EU—summary of a Focus group discussion

**DOI:** 10.3389/fmed.2025.1593644

**Published:** 2025-07-10

**Authors:** Stiina Aarum, Claudia Popp, Alexa Hunter, Miha Skvarc, Ilona Reischl, Hilke Zander, Joerg Zinserling, Petra van Leeuwen, Jonathan Sutch, Tim Chesworth, Elisabeth Kapeller, Andreas Emmendoerffer, Iordanis Gravanis, Christelle Bouygues, Claudia Vincenzi, Michael Berntgen

**Affiliations:** ^1^European Medicines Agency, Amsterdam, Netherlands; ^2^F. Hoffmann-La Roche Ltd., Drug Regulatory Affairs, Basel, Switzerland; ^3^Merck KGaA, Darmstadt, Germany; ^4^Austrian Medicines and Medical Devices Agency (AGES MEA), Vienna, Austria; ^5^Paul-Ehrlich-Institut, Langen, Germany; ^6^Biostatistics and Special Pharmacokinetics Unit, Bundesinstitut für Arzneimittel und Medizinprodukte (BfArM) (Federal Institute for Drugs and Medical Devices), Bonn, Germany; ^7^Team-NB, DEKRA Certification B.V., Arnhem, Netherlands; ^8^Team-NB, BSI, Milton Keynes, United Kingdom; ^9^AstraZeneca ORSSE, CMC Regulatory Affairs, Macclesfield, United Kingdom; ^10^Sandoz GmbH, Kundl, Austria; ^11^F. Hoffmann-La Roche Ltd., Pharma Technical Regulatory, Basel, Switzerland

**Keywords:** development, combination products, IVD, Medicines, advice, innovation, devices

## Abstract

Although there is growing use of medicinal products in combination with medical devices, including *in vitro* diagnostics (IVDs) and software as a medical device, their development in the EU is proving to be more challenging due to the complexities in working across the regulatory frameworks for medicinal products, medical devices and IVDs. One of the concerns is the lack of a multistakeholder platform [European Medicines Agency (EMA)/Scientific Advice Working Party (SAWP); National Competent Authorities (NCAs) in charge of medicinal products; NCAs for medical devices; notified bodies (NBs)] for scientific and regulatory advice on medicinal products used in combination with medical devices or IVDs [including companion diagnostics (CDx)]. A multistakeholder platform would allow for an opportunity to discuss the scientific expectations of the different decision makers involved and guide the identification of an appropriate development path for both medicine and medical device/IVD (including CDx). In search of a pragmatic approach to facilitate discussions on evidence generation plans, it was agreed in the “9th EMA-Industry stakeholder platform on research and development support” meeting in December 2022 to set up a dedicated Focus group to determine the possibility of provision of scientific advice for medicinal products in combination with a medical device or IVD (including CDx). The Focus group had participants from the EMA SAWP, the NCAs, the NBs, and industry and the EMA. The group explored what kind of scientific questions would benefit from being addressed in comprehensive discussions on evidence generation planning in a multistakeholder setting, and who would be the required decision makers and experts for such multidisciplinary discussions. The discussions covered nine study cases: four were drug-device combinations and five were drug—IVD/CDx combinations. The scope of the discussions excluded stand-alone high-risk medical devices and IVDs for which there was an ongoing scientific advice pilot involving the medical device expert panels. In addition, low-risk stand-alone medical device/IVD developments were also out-of-scope. This article presents the insights of the discussions on the nine study cases reviewed by the Focus group and explores options for next steps to inform future policy and technical discussions on these innovative healthcare developments.

## 1 Introduction

The European Union (EU) has introduced significant steps to ensure the safety and performance of medical devices and *in vitro* diagnostic (IVD) medical devices within its Member States. At the heart of this effort lie the regulatory frameworks established by EU Regulation 2017/745 (the Medical Devices Regulation or MDR) and EU Regulation 2017/746 (the *In Vitro* Diagnostic Medical Devices Regulation or IVDR). These regulations mark a comprehensive overhaul of the previous directives governing medical devices and IVDs, with the goal of enhancing patient safety, streamlining regulatory processes, and adapting to technological advancements in the healthcare industry (1, 2).

This project looked at the type of scientific questions that could benefit from a multistakeholder scientific dialogue when developing medicinal products used in combination with medical devices. Although there is no legal definition of combination products in the EU apart from integral drug-device combinations, these are understood to also cover medicinal products that are co-packaged with a medical device or those where the use of a specific medical device is referenced in the product information of the medicinal product (3, 4). The latter also includes the use of an IVD/companion diagnostic (CDx). Under European regulations, a CDx is an *in vitro* diagnostic medical device which is essential for the safe and effective use of a corresponding medicinal product. The CDx identifies patients who are most likely to benefit from a treatment or who are at risk of serious adverse reactions as defined in Article 2(7) of the IVDR, i.e., a CDx is used to assess the suitability of a patient for a targeted therapy (2).

Currently, scientific advice on medicinal products is provided by both the NCAs in charge of medicinal products at national level and the EMA’s Committee for Medicinal Products for Human Use (CHMP) based on the recommendation of the Scientific Advice Working Party (SAWP), at the European level (5). As such, scientific advice may be requested for all medicinal products for use in humans, [as defined in Directive 2001/83 83 (as amended)], irrespective of the eligibility of the medicinal product for the centralised procedure, on aspects of the design of studies, trials and programs to support quality, safety and efficacy of the medicinal product. For medicinal products with an orphan designation, Article 6 of the Regulation on Orphan Medicinal Products (EC) 141/2000 entitles the sponsor of an orphan medicinal product to request advice (Protocol Assistance) from the Agency on the conduct of the various tests and trials necessary to demonstrate the quality, safety and efficacy of the medicinal product (6).

Scientific advice for medical devices on the other hand, is provided by some NCAs in charge of medical devices while other NCAs limit themselves to borderline issues and keep similar restrictions to medical devices as notified bodies (NBs), who are not allowed by law to provide advice on their development. In addition, the EU Innovation Network offers Innovation Task Force (ITF) meetings, which can also be attended by NCAs with competency on medicinal products and medical devices, providing a forum for early dialogue with applicants on innovative aspects in medicines development, including emerging therapies and technologies (7). In addition, the EMA has recently conducted two pilots to offer scientific advice on the intended clinical development strategy and proposals for clinical investigation to certain high-risk medical devices and orphan medical devices (8). In the high-risk medical devices pilot, advice was provided by the medical device expert panels, and the scope was limited to all class III devices and class IIb active devices intended to administer and/or remove medicinal product(s). Recently, a regular procedure for scientific advice on these types of high-risk medical devices was established by the EMA (9).

NBs are not involved in the development phase, including approval of clinical studies for MDs, nor in providing scientific advice. Annex VII Section 1.2 of the MDR and IVDR outlines the requirements for independence and impartiality for a NB and activities such as consulting are prohibited under the legislation (1, 2).

Article 117 of the MDR introduces requirements to involve a NB at the time of marketing authorisation of a medicinal product forming an integral part with a medical device, to assess conformity with the General Safety and performance requirements as defined in Annex I MDR (1). Of note, Article 117 MDR does not apply to combined advanced therapy medicinal products (ATMPs) and a dedicated consultation procedure is foreseen in the ATMP regulation (EC) 1394/2007. There have not been examples of the application of this article in marketing authorisations for an ATMP but products currently under development make them likely for the future. The topic of combined ATMPs is not further addressed or discussed in this article.

The IVDR establishes a new connection between the assessment of a CDx and the corresponding medicinal product. It mandates that the NB in charge of the CDx certification seeks a scientific opinion from the competent authority responsible for authorisation of the medicinal product (NCA or EMA) regarding the suitability of the CDx for use with the relevant medicinal product(s) before granting an EU conformity assessment certificate. This interaction involves directly or indirectly several stakeholders (medicines regulators, NBs, IVD developers, and medicines developers) and it has been highlighted that there is a need for increased collaboration and alignment of assessments performed by different stakeholders in this process (10).

In the EU, the Medical Device Coordination Group (MDCG) deals with key issues from the medical devices sector and the MDCG guidance documents present a common understanding of how the MDR and IVDR should be applied in practice aiming at an effective and harmonised implementation of the legislation.

For the NBs, MDCG 2022-14 (Transition to the MDR and IVDR—Notified body capacity and availability of medical devices and IVDs) encourages NBs and manufacturers to organise structured dialogues before and during the conformity assessment process aimed at regulatory procedures where this is useful to enhance the efficiency and predictability of the conformity assessment process, while respecting the independence and impartiality of the notified body and it states that such dialogues should not be considered consultancy service (11, 12). It is currently not possible for NBs to provide advice on medical devices development.

Development of medicinal products used in combination with medical devices/IVDs (including CDx) involves questions surrounding both the separate entities and unique aspects of their combined use and may also require additional specific expertise, i.e., questions related to the scientific validity, analytical performance and clinical performance including cut-off value selection or topics related to the development of digital health technologies. Currently, there are only a few specific EU guidelines regarding the data collection during development to generate appropriate evidence for parallel authorisation/certification. Developers have expressed concerns that the new regulatory interface between the pharmaceutical regulatory framework and MDR/IVDR is burdensome and might hinder innovation and conduct of clinical trials in the EU, and thereby ultimately cause delays in market access. Furthermore, there is currently no single entry nor a joint platform to discuss the regulatory pathway and development from the scientific perspective with different stakeholders in the EU such as NCAs, NBs, developers and the EMA.

In this article we present and evaluate study cases based on the discussions of the Focus group organised by the EMA to understand what kind of scientific questions would benefit from a multi-stakeholder scientific advice involving device bodies and the EMA and who would be the required experts to discuss and provide feedback on the different questions.

## 2 Materials and methods

Following discussions at the 9th EMA Industry stakeholder platform on research and development support meeting in December 2022, a Focus group with members from the SAWP, experts from the NCAs in charge of the evaluation of medicinal products used in combination with medical devices or IVDs as well as those involved in the CDx consultation procedure, industry, NBs and the EMA was established with the intent to eventually set-up a multistakeholder scientific advice pilot. The members were invited to propose cases where combined development would benefit from a joint scientific discussion by multiple stakeholders. Questions on the clinical trial and performance study authorisation and conduct, as well as procedural and regulatory questions were not in scope for this exercise. Questions on device classification were also considered out of scope.

The Focus group members worked in two parallel break-out sessions, one for drug-device combinations and another for drug-CDx combinations, to:

- Perform a comprehensive analysis of types of questions which can be addressed in scientific advice for each specific case.
- Identify relevant stakeholders and experts that would be required for multidisciplinary discussions in the context of such scientific advice procedures and opportunities/restrictions for their participation.

Due to the restricted involvement of the NBs in the development phase, it was not possible to envisage a multistakeholder scientific advice pilot which includes NBs. This publication presents the results from the analysis of the scientific questions and the proposed experts to discuss them for each of the cases.

## 3 Results

The Focus group selected nine study cases for further evaluation; four cases were drug-device combinations (Table 1) and five were drug-IVD/CDx combinations (Table 2). Eight of the cases were proposed by the industry and one case was proposed by the SAWP. Below we summarise each study case:

**Table 1:** Overview of the drug-device combination cases.

Development stage	Possible question(s)	Proposed stakeholders
Pre-marketing authorisation phase: co-packaged product—stability strategy	Alignment on the stability plan of the final DDC presentation which has multiple constituents, including leveraging device accelerated aging data and setting shelf life based on the shortest expiration period	EMA, NCA, NB
Pre-marketing authorisation phase: On-Body Delivery System—development and registration strategy	Questions on clinical trial design and requirements of device-related endpoints as well as bridging from PFS to OBDS Technical aspects of control strategy, verification testing representing intended use and expected stability conditions for shelf life studies Obtain understanding of the alignment and connection between: The conformity assessment requirements for the OBDS medical device and The MAA assessment of the medicinal product with which the OBDS is to be co-packaged	EMA, NCA, NB
Pre/Post-marketing authorisation phase: Clinical development: development of an app classified as MDSW (Medical Device Software) to be used in combination with a medicine to guide titration	Questions to the clinical strategy and data needed for the CE mark of the MDSW solution as well as the evidence needed to be licensed together with the medicine and if these can be obtained through a combined process	EMA, NCA, NB
Post-marketing authorisation phase: Line extension of an already authorised medicinal product to add a new delivery method	Questions on the bridging strategy to demonstrate performance equivalence between the different versions of the autoinjector used in the development	EMA, NCA, NB

**Table 2:** Overview of the drug-CDx combination cases.

Development stage	Possible question(s)	Proposed stakeholders
Pre-marketing authorisation phase	Analytical and clinical performance plan & related CT design aspects to support both, medicinal product (MP) MA and CDx CE-marking Cut-off values & demonstration of scientific validity (e.g., for novel CDx) Requirements for follow-on CDx (i.e., bridging studies, analytical performance, choice of reference device, interchangeability and different performance)	EMA, NCA, NB
Labelling of the MP	Early dialogue useful to discuss label implications based on expected magnitude of benefit and proposed study design	EMA, NCA, NB
Orphan development	Issues are magnified for orphan medicinal product—CDx combinations (limited patient population, ev. single arm trial design etc.)	EMA, NCA, NB
Bridging cases e.g., More than one CDx is used in CTs IVDD CE marked CDx becomes an investigational CDx (for future CE marking under IVDR) Local testing was initially used prior to CDx development CDx developed only outside EU	Design/plan for bridging studies to generate required analytical and (if needed) clinical performance data for the “final” CDx to ensure consistent input according to different stakeholders’ perspective and remit: MD: data requirement to support performance of final CDx & leveraging data from previous CDx MP: requirements to support the robustness of data in support of benefit/risk evaluation of the MP	EMA, NCA, NB
Post Marketing Authorisation Phase	All questions addressed under above cases	EMA, NCA, NB

### 3.1 Cases of medicinal products used in combination with devices

#### 3.1.1 Medicine used with a co-packaged medical device—stability strategy

This case explored the types of questions that would be relevant for an aligned stability plan of a medicinal product and a co-packaged medical device in the pre-authorisation phase. In general, the stability strategy of a co-packaged drug-device combination was considered a suitable topic for multi-stakeholder scientific advice.

Questions viewed as applicable included how to seek endorsement of a deviation from International Conference for Harmonisation (ICH) guidelines [in particular ICH Q1A (R2) Stability testing of new drug substances and drug products—Scientific guideline] for the drug product stability strategy, and which of the device functionalities should be incorporated in the ICH Q1A stability program for the co-packaged drug-device product (13). Additionally, when considering the stability strategy for the device component of the co-packaged product, the possibility of leveraging device accelerated ageing data and setting a shelf life based on the shortest expiration period could also be explored in a multi-stakeholder scientific advice procedure.

Questions could also extend to the design of a clinical trial that would provide data for both the medicinal product for regulatory decision making and the appropriate device-related endpoints for the CE-mark, and to know if separate studies would be required. The questions for this type of combined development may also include technical questions on the development of a device. However, NBs currently have no remit to answer these questions, and therefore it is unclear where or at which platform such topics could be discussed.

#### 3.1.2 On Body Delivery System—development and registration strategy

The addition of an On-Body Delivery System (OBDS) to an already approved pre-filled syringe (PFS) presentation was identified as another example of a drug-device combination that would benefit from multistakeholder scientific advice during the development phase. In most cases the device manufacturer of an OBDS adapts the device for the respective medicine (which, for example, is contained in a cartridge). Therefore, irrespective of whether the OBDS forms an integral product with the cartridge or is co-packaged, input from the different stakeholders and scientific advice, including technical and clinical questions, is considered suitable in the following context:

a) To discuss the design of the clinical trial (medicine) and clinical investigation (device) with respect to clinical endpoints, device functionalities, number of patients needed for establishing the functionality and performance of the device, and data to support a bridging strategy between the PFS and the OBDS.
b) To obtain feedback on relevant requirements applicable to this case for the CE-marking process of the OBDS and for the marketing authorisation application (MAA) assessment process of the medicine with which the OBDS will be co-packaged. However, NBs cannot reply to questions on how to comply with requirements. While the manufacturer can exchange with the NB in the pre-submission phase of the technical documentation, this is a challenge that the interaction between the applicant and the NB only starts close to the filing of the application for the conformity assessment when the development of the device is completed, while this example is related to an early development stage, prior to filing an application.
c) To define the device control strategy, verification testing for the intended use, and expected stability conditions for shelf-life studies. In addition, EMA noted that drop test and accelerated ageing questions could also be discussed during the scientific advice procedure.

#### 3.1.3 Medical Device Software—clinical development

This case concerned a non-integral app qualified as a Medical Device Software (MDSW) which is to be used in combination with a medicine to guide titration. The titration is guided by a software algorithm, leading the patient to either decrease or increase the dose of a medicine.

Questions for scientific discussion focused on the clinical development strategy and data needed for the drug MAA and the conformity assessment of the MDSW solution, and the evidence needed for them to be licensed together. The following questions/topics were also deemed appropriate for multi-stakeholder scientific advice:

a) If a combined clinical study design (including endpoints) could be an option, and, if yes, what the requirements would be for such a clinical study?
b) To evaluate the methodologies needed to ensure that data from the digital solution can be used to support a MAA.
c) Requirements for developing MDSW either to be co-developed with a new medicine or to be added to a label of an existing medicinal product.

Questions were raised by the Focus group to clarify if the primary intended use of the device is linked to the medicine, e.g., is it possible to use the medicine alone or only in combination with the device and if the MDSW would then be fully integrated in the titration device or if the MDSW and the titration device would be considered two separate devices.

Comments were made by the medicines’ regulators that the number of studies needed to answer the questions may need discussion. NBs highlighted that the MDR does not require that clinical data comes from a device investigation only, but the sponsor would need to submit an application for a clinical investigation for the device in the clinical trial. Taken together, the possibility of a combined study could be a suitable topic for a scientific dialogue.

It was also discussed if the device could possibly serve as a platform model to be used with a group of medicines, and The Focus group learnt that there is potential for a platform, but at this point the development is focused on use with one medicine only and the question is how/what clinical data needs to be gathered to allow the app to be used in combination with a medicine to guide titration.

The NBs questioned if the algorithm is new or whether it has been used in other settings and/or is known from the literature, and what kind of clinical data is expected. It was clarified that the algorithm is new but that the safe posology range of the medicine is based on existing knowledge set by clinicians.

EMA raised a question on who will look at the acceptability of the range and if there will be a requirement for a dose range in the algorithm. This was identified as a potentially controversial topic that would also benefit from further discussion.

It was agreed that the presented questions would benefit from a multistakeholder discussion.

#### 3.1.4 Line extension of a medicine for a post-marketing change to introduce a pre-filled syringe to an autoinjector

During the development, several versions of the autoinjector have been used. In the scientific advice, the sponsor sought endorsement of the proposed bridging strategy to demonstrate performance equivalence between the different versions of the autoinjector used in the development.

This case explored a scenario where an auto-injector has been updated three times during its development, and a bioequivalence (BE) study conducted with an earlier version of the autoinjector, did not conclude equivalence with the approved PFS presentation. The Focus group reviewed how the multistakeholder platform would be beneficial in reviewing the bridging strategy to demonstrate performance equivalence among the different autoinjector versions. In this discussion, it was highlighted that there is a need to understand the reasons for the negative BE study.

For the technical aspects, it was agreed that a joint scientific dialogue to understand the level of review of the different versions of the autoinjector by the EMA/NCAs, and NBs would be appreciated. Industry pointed out that currently there is no place to go to discuss performance evaluation and thus this would be positive.

### 3.2 Drug-CDx combination cases

#### 3.2.1 Pre-marketing authorisation phase: biomarker validation and combined study design to support approval

This case study looked at the evidence required to support the validity of the predictive biomarker (including its analytical and clinical performance and cut-off value selection) in relation to the clinical study design to support the approval of the medicinal product and certification of the CDx.

Questions related to the scientific validity, analytical performance and clinical performance, including cut off value selection, were overall considered relevant for multistakeholder input (SAWP/CHMP, IVD and medicines regulators and NBs). Contribution from NBs was considered of relevance for aspects related to analytical and clinical performance, while their input may not be systematically needed for other aspects, such as discussion on cut-off values or scientific validity. For the latter two topics, NB contribution may be provided on a case-by-case basis, e.g., may vary depending on the type of development program, e.g., orphan medicines vs. non-orphan medicines and specific questions posed.

NB contribution was considered especially useful for follow-on CDx questions on topics including bridging studies such as analytical concordance, acceptable differences in performance parameters, choice of reference device, and interchangeability (refer also to case study 4).

While it was recognized that there are important questions which would benefit from further discussion/clarifications with regards to clinical trials and performance study authorisation and conduct (e.g., evidence needed prior to clinical trial start, need for performance study or not, requirements to support an IVDR performance study authorisation, conduct of performance study within/as part of a clinical trial), it was agreed that these kind of questions would fall under the remit of NCAs reviewing and approving the clinical trial and performance study applications. This is now being looked at in the COMBINE project (8).

Overall, it is acknowledged that NBs cannot discuss aspects related to the performance evaluation plan (including combined performance studies and clinical trials) in the development phase. Industry also highlighted that it is not uncommon that different feedback on performance studies is received at national level.

#### 3.2.2 Drug labelling

This case discussed potential labelling questions for the medicine Summary of Product Characteristic (SmPC) after readout of a pivotal trial using an investigational device/CDx.

The Focus group considered that some specific scenarios concerning label implications could be discussed as part of a multistakeholder scientific advice procedure during the pre-authorisation phase. This could concern, for example, questions on the design of the study, hypothesis testing and expected magnitude of effect. This could be of particular relevance where limited data are expected to be available, e.g., for products developed in rare disease settings. However, in general it was considered more appropriate to discuss the impact of the available study results and the impact on the label/target population during the EMA MAA pre-submission meeting.

### 3.3 Orphan drug-CDx combination developments

This case sought to understand the evidence generation requirements for an orphan drug-CDx combination, at the stage of pivotal trial design development.

Overall, the Focus group considered that the general points discussed earlier in terms of analytical and clinical performance were also applicable in this case, while acknowledging the unique challenges associated with orphan developments, where a limited number of patients is available for inclusion in clinical trials due to the rarity of the disease, and/or a single arm trial is considered in certain cases. These limitations in evidence generation, and their associated uncertainties, will need to be discussed during medicinal product and medical device/IVD data-generation planning.

Furthermore, it was clarified that there is currently no legal definition of “orphan devices,” and these devices must therefore meet the same standards as any other devices. While it was acknowledged that development of devices used in orphan conditions would benefit from further discussion by relevant stakeholders, it was noted that, in-house testing may satisfy some of the evidence requirements needed to support these developments. For in-house tests in particular, it was clarified that relevant safety and performance requirements are available in Annex I of the IVDR. The implications of local testing on the safety and efficacy of the medicine would also need to be considered.

### 3.4 Bridging strategies

This case sought alignment on bridging strategies to generate required evidence for CE certification in cases where more than one device or diagnostic platform was used in a clinical trial also intended to support a MAA for the medicine.

Several scenarios were discussed:

a) More than one device is used in clinical trials with a CDx:

The Focus group considered that the bridging strategy to the final “to be marketed” CDx needs to be discussed from several angles, including (1) data requirements to support the performance of the final “to be marketed” device, and to which extent data from the previous device can be leveraged to support the performance of the subsequent/final device and ultimately certification, and; (2) requirements to ensure the robustness of the data presented in support of the benefit/risk evaluation of the medicinal product.

b) IVD with a CE certificate under the *in vitro* diagnostic directive (IVDD) to be bridged with an investigational device for future CE marking under IVDR:

It was noted that an IVD CE marked under IVDD requires additional data/documentation to meet safety and performance requirements under IVDR. The group also acknowledged that every device is assessed on its own and not necessarily compared to (an)other device(s). Furthermore, it was clarified that analytical performance does not directly translate into clinical performance which requires additional evidence.

c) Bridging strategy in case local testing was initially used prior to CDx development in the clinical trial:

Confirmation of local testing in the course of the clinical trial using the intended CDx was recommended, e.g., with parallel central testing. The topic was considered to have several dimensions: from a medicinal product perspective the impact on the robustness of data requires planning and discussion, while from a medical device perspective it should be discussed whether the data would be sufficient to support a CDx claim in accordance with IVDR.

Since analytical performance is expected to be a key aspect of bridging strategies and clinical data may only be rarely provided, the overall conclusion of the Focus group was that contribution by MD bodies would be beneficial to advise on these types of questions from an IVD requirements perspective to complement the medicines regulators’ advice.

Overall, the group considered that questions on bridging strategy were relevant for multistakeholder input. However, considering the different stakeholders’ remits, the Applicant should present their questions and objectives clearly to ensure relevant feedback is received.

It was broadly acknowledged that the use of various devices adds significant complexity to clinical trial approval and conduct. Although not discussed in the Focus group in detail, another important example of a complex CDx platform that needs to be considered for innovative treatments is Next Generation Sequencing (NGS). NGS holds great promise and can play a crucial role in identifying genetic alterations relevant to treatment decisions. Specifically, it can detect mutations in many tumour genes in one analysis, offering a more comprehensive test compared to single-gene tests. The challenge comes when integrating NGS into a CDx use/development. Providing evidence of accuracy, reproducibility, scientific validity and clinical performance of the NGS-based CDx, that analyses multiple genes in a single test and is used to identify a drug tailored to a patient’s genetic profile, is difficult. For example, validation of individual genomic alterations in accordance with IVDR would profit from scientific advice discussions.

### 3.5 CDx development for new indications—post marketing authorization stage

This case explored questions relating to the regulatory requirements and CDx development strategy for a new indication, i.e., before the pivotal trial had started.

The Focus group highlighted that considerations on bridging related to the first topic constitute the main issues relating to this point, as well.

## 4 Discussion

Healthcare innovations are increasingly at the interface of medicines and technology such as medical devices, IVDs, and digital health tools. In this article, we illustrate a variety of examples of medicines that are in development in parallel/jointly with such technologies. The results highlight the need for increased scientific dialogue between the stakeholders as well as the potential for increased collaboration within the evolving regulatory framework throughout the life cycles of the medicines and medical devices/IVDs.

The EU regulatory landscape has different frameworks for medicinal products, medical devices and IVDs, and for the development of drug-device/CDx combinations, MDR and IVDR must be considered in conjunction with the pharmaceutical legislation. The combined development also involves many different stakeholders: EMA, NCAs, NBs, pharmaceutical industry and MD and IVD developers, and overall, the regulatory environment is perceived as highly complex and difficult to navigate and predict.

The COMBINE project, launched by the European Commission and the Member States, has analysed the challenges at the interface of MDR/IVDR/CTR and has now entered into its second phase to find possible solutions. While the project focuses on the individual authorisation processes of clinical trials of medicinal products, clinical investigations of medical devices and performance studies of *in vitro* diagnostics at the national level, the results also show that 57% of Member State competent authorities offer advice to sponsors of combined studies prior to application. However, only 11% offer national scientific advice on aspects other than clinical trials. While some options are available to receive scientific/technical advice, sponsors and manufacturers report difficulties in getting advice, and the consistency and reliability of advice given (14). In our study, we present cases with specific questions for which options for scientific dialogue between the EMA, NCAs, NBs and the developers are currently limited and which would benefit from improved interaction opportunities with relevant stakeholders.

Our findings are in line with previous publications that looked at the regulatory environment specifically for CDx and digital health tools development, respectively (10, 15). Vebaanderd et al. (10) described the European regulatory framework for certification of CDx and analysed challenges for medicine and CDx co-development, highlighting the need for not only legal hurdles to be overcome but also the importance of a close dialogue between the involved stakeholders. Colloud et al. (15) focused on case studies using digital health technology tools used throughout the lifecycle of medicinal products, starting from their development, and illustrated the complexity of the interactions specifically for these combinations; they stressed the unavailability of a pathway that would allow for a formal joint or parallel advice from medicine regulators and device bodies that may result in a slower uptake and development of digital health technology tools.

As an exploratory step to overcome these challenges, the Focus group findings specify cases with a variety of scientific questions, and indicate experts between whom more dialogue, together with scientific guidance, would be needed to expedite development in the EU of innovative drug-device combinations and medicinal products using CDx. The presented cases show that the need for a multistakeholder scientific advice relates to questions on development strategies throughout the life cycle of medicines used in combination with medical devices and/or CDx, and although the possible expert needs depend on the wording of the question, for the majority, the proposed stakeholders are the EMA, NCAs, Industry and NBs. The developers would need to present their questions and objectives clearly, considering the different stakeholders’ remits.

While the Focus group discussions also recognised that there are important questions which would benefit from further discussion and clarifications with regards to clinical trials and performance study authorisation and conduct (e.g., evidence needed prior to clinical trial start; need for performance study or not; requirements to support an IVDR performance study authorisation; conduct of performance study within/as part of a clinical trial), it was agreed that these kind of questions would fall under the remit of NCAs reviewing and approving the clinical trial and performance study applications, and these were not further discussed by the group. These questions are now being looked at in the COMBINE project (14). The Clinical Trial Coordination Group (CTCG), MDCG and IVD Working Groups are also developing questions & answers, with the intention to publish regular updates based on questions received. However, these cover general principles and there is currently no possibility for a multistakeholder product-specific advice in this regard. Structured interaction opportunities with relevant stakeholders are needed and should be further considered.

Further in the discussions within the Focus group, however, it became clear that NBs cannot currently participate in scientific advice due to legal restrictions, as they are not allowed to consult. NBs and manufacturers are instead encouraged to organise structured dialogues before and during the conformity assessment process and it is understood that these dialogues should not consist of consultancy service. While the recent update from MDCG on the scope of structured dialogue provides clarification, it remains that NBs cannot provide advice on device development (12).

The use of medicinal products in combination with medical devices is growing. Industry stakeholders have highlighted the access to relevant experts and harmonised approaches as the key to foster innovation in the EU, and to finally ensure that these innovations reach European patients rapidly and safely (16). The importance of these innovative healthcare solutions for the competitiveness of Europe was also recently highlighted in the report by M. Draghi who suggests to streamline the set-up and management of multi-country trials in the EU by establishing rules to address challenges for studies which combine medicines with medical devices and the application of AI (17).

A limitation to this work is that no case concerning a medicine used with an AI-enabled medical device was included. However, it is expected that the same type of interactions as described herein, would also be relevant for such combinations and there is no reason to exclude AI-enabled devices from eventual future considerations on multistakeholder scientific advice. The joint Heads of Medicines Agencies (HMA)-EMA Network Data Steering Group (NDSG) focuses on data that the European medicines regulatory network receives, analyses or offers advice for, and has the strategic goal to leverage data and AI in regulating human and veterinary medicines in the EU (18). Also, one of the key dimensions in the AI workplan to 2028 is continuous support to products in development as well as the development and evaluation of appropriate guidance for the use of AI in the lifecycle of a medicine (19).

It is acknowledged by all Focus group members that more dialogue and regulatory expertise sharing is needed to allow that the innovative combined developments with the fast-paced advances of technologies have a solid regulatory framework that supports innovation while also safeguarding the patient safety.

## Author’s note

MS is a seconded national expert from Hospital Golnik—University Clinic of Pulmonary and Allergic Diseases, Golnik 36, 4204 Golnik, Slovenia.

## Data Availability

The datasets presented in this article are not readily available because the datasets are presented in the manuscript.

## Appendix: Glossary

### CDx—companion diagnostics

A companion diagnostic is defined in Article 2(7) of Regulation (EU) 2017/746 (*in vitro* diagnostic medical device regulation, IVDR) as follows:

“Companion diagnostic” means a device which is essential for the safe and effective use of a corresponding medicinal product to:

- Identify, before and/or during treatment, patients who are most likely to benefit from the corresponding medicinal product; or
- Identify, before and/or during treatment, patients likely to be at increased risk of serious adverse reactions as a result of treatment with the corresponding medicinal product.

### In-house IVD

An IVD manufactured and used within the same health institution as outlined in IVDR Article 5(5). Health institution is defined in IVDR Article 2(29), as defined in MDCG 2022-10 (20).

### Combined studies

Studies that involve:

- A clinical trial of a medicinal product in parallel with a performance study of an in vitro diagnostic.
- A clinical trial of a medicinal product in parallel with a clinical investigation of a medical device.

### “COMBINE” project

The COMBINE project aims to analyse the root causes of the challenges encountered by sponsors in conducting combined studies and identify possible solutions to these challenges (14).

### DDC—Drug Device combinations

Combination of medicinal product and delivery device, see also Article 1(8) and 1(9) MDR.

### EMA scientific advice

At any stage of a medicine’s development, a developer can ask guidance and direction from EMA on the best methods and study designs to generate robust information on how well a medicine works and how safe it is, regardless of whether the medicine is eligible for the centralised authorization procedure or not. EMA scientific advice is given by the CHMP on the recommendation of the SAWP except in the case of medicines that are intended to treat, prevent or diagnose a disease causing a declared public health emergency, for which scientific advice is given by the CHMP based on recommendation of the Emergency Task Force (ETF) (21).
